# A Health Systems Strengthening Approach to Address the High Burden of Hepatitis C in Pakistan

**DOI:** 10.1111/jvh.14050

**Published:** 2024-12-21

**Authors:** Mahnoor Qureshi

**Affiliations:** ^1^ School of Health Sciences The University of Manchester Manchester UK

**Keywords:** blood‐borne pathogens, cross infection, delivery of healthcare, developing countries, endemic diseases, hepatitis C, Pakistan, primary health care, task shifting

## Abstract

Hepatitis C virus infection is a serious liver disease that can progress to cirrhosis and, in chronic cases, lead to liver cancer or liver failure. Pakistan has the second highest burden of HCV in the world, a rising number of liver cancer cases and a unique pattern of healthcare‐associated HCV transmission. Unfortunately, the country is not on track to meet the WHO's target of complete elimination of HCV by 2030. The current reliance on vertical programmes for hepatitis elimination may seem effective in the short term, but is often unsustainable, ineffective and contributes to the fragmentation of the health system. This review proposes a health system strengthening approach to HCV detection and prevention in the country. It critically evaluates the country's health system and the existing evidence on HCV prevention and treatment, proposing evidence‐based strategies for decentralising HCV services and integrating them into the primary healthcare infrastructure. It examines the effectiveness of methods such as task shifting and targeted interventions while suggesting changes to healthcare practices to reduce healthcare‐associated transmission of HCV and other blood‐borne pathogens.

## 1 Introduction

Hepatitis C virus (HCV) is a viral infection that affects the liver, initially causing mild to no symptoms among infected individuals [1]. If left untreated, HCV can lead to liver disease and occasionally cirrhosis. In some cases, patients with cirrhosis will develop serious complications, such as liver cancer or liver failure [2]. The World Health Organization (WHO) estimates that in 2019, 58 million people worldwide were living with HCV, and 1.1 million died due to the infection and its effects [3].

**FIGURE 1:**
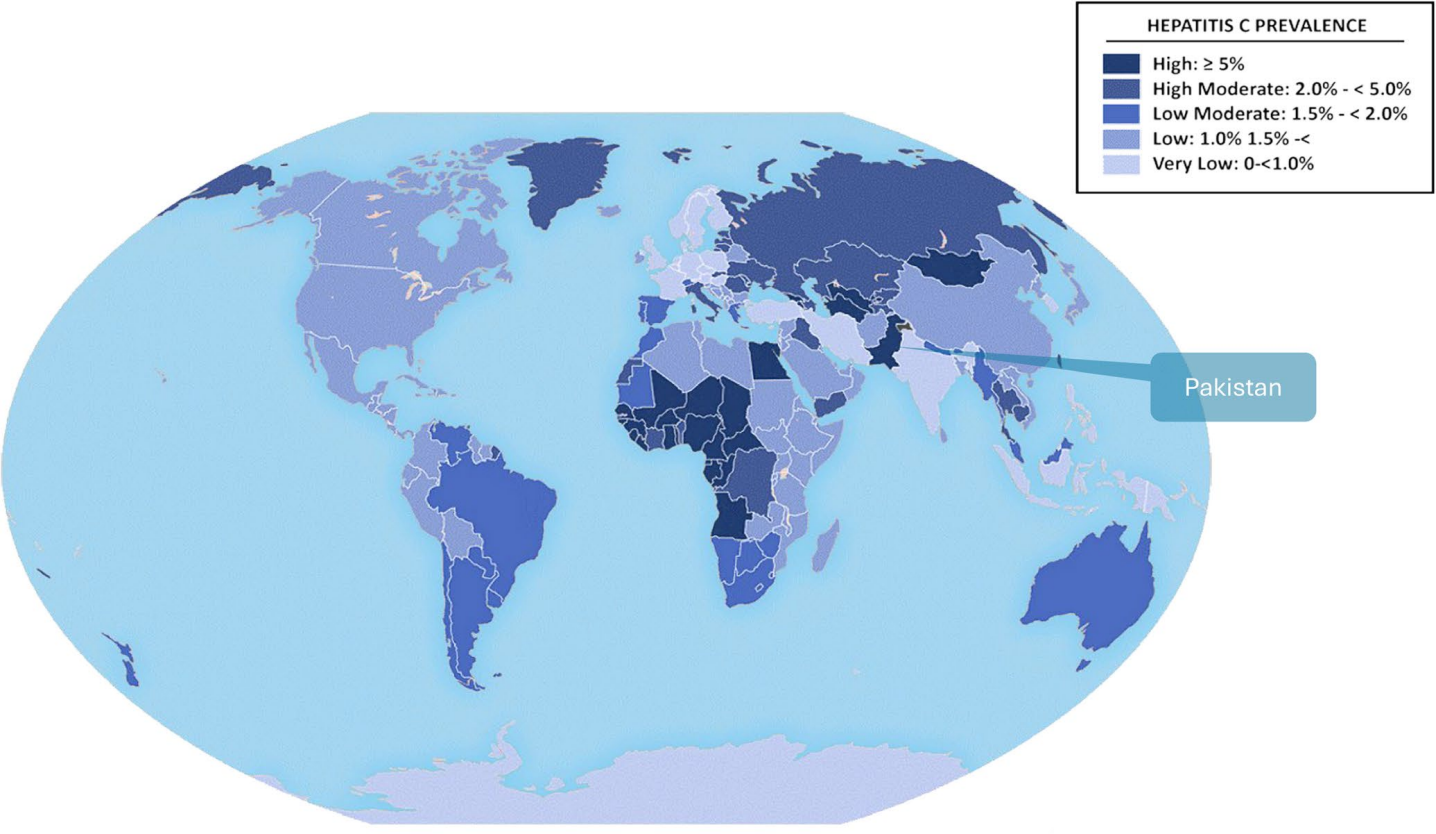
Worldwide prevalence of Hepatitis C. It is evident that Pakistan has a very high rate of infection (≥ 5%) compared to other countries in the region, such as Iran and India. Data obtained from the CDC website [6].

Pakistan has the second highest burden of HCV infection in the world, with 9.8 million people (4.3% of the population) living with HCV [4] (Figure 1). The country reported 461,000 new chronic HCV infections in 2019 [5], and has a rising number of liver cancer cases [2]. In 2019, 17,644 people (CI: 12,752–24,554) died of hepatitis‐related causes in Pakistan [4]. Global HCV elimination depends on the proportion of cases identified and treated in high‐burden countries, such as China, Egypt and Pakistan [1]. This paper will examine the burden of HCV in Pakistan, evaluate the strengths and weaknesses of the country's health system in eliminating HCV, and propose evidence‐based strategies for prevention, testing and treatment.

### 1.1 Prevalence of HCV in Pakistan

**FIGURE 2:**
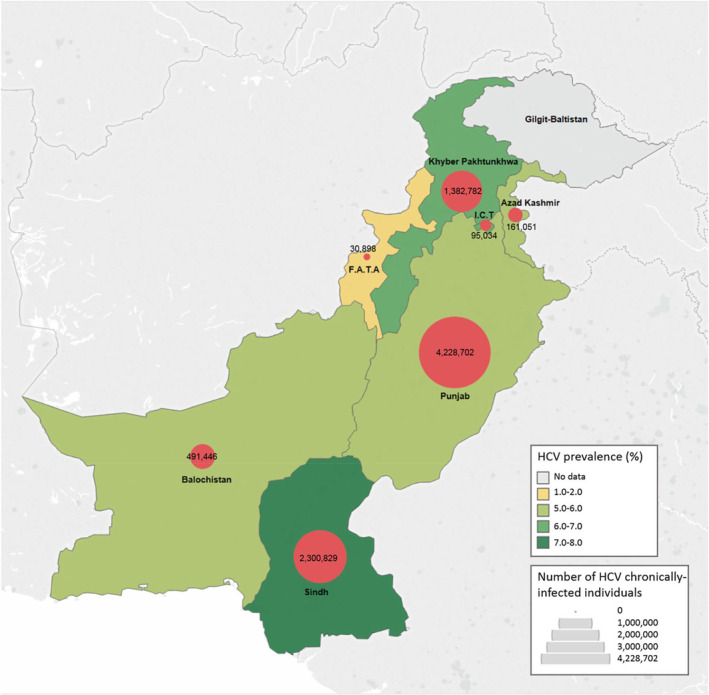
Map of the province‐wise prevalence of HCV and distribution of HCV chronically infected individuals in Pakistan. Sindh has the highest prevalence (7%), followed by Islamabad Capital Territory (ICT; 6.9%), Khyber Pakhtunkhwa (KPK; 6.6%), Azad Kashmir (5.8%), Balochistan (5.8%), Punjab (5.6%) and Federally Administered Tribal Areas (FATA; 0.9%). No data are available for Gilgit Baltistan and Indian occupied Kashmir. Adapted from Mahmud, Al Kanaani, Abu‐Raddad [7].

Mahmud, Al Kanaani, Abu‐Raddad [7] created meta‐regression models for HCV prevalence across different populations in Pakistan (Figure 2). Their findings show minor differences in prevalence across provinces [7], which contradicts the 2007–2008 national survey that reported more significant variations [8].

As in much of the developing world, HCV transmission in Pakistan is mainly healthcare‐associated, spread via inadequately screened blood transfusions [9] and improperly sterilised invasive medical devices [10]. Thus, high‐risk clinical populations, such as patients with thallasaemia, haemophilia and those requiring haemodialysis, are at an increased risk of HCV infection (AOR: 7.8, CI: 4.8–12.7) [7] due to frequent exposure to blood transfusions or medical injections [11].

Unsafe medical practices are very common. Pakistan has the highest rate of therapeutic intramuscular injections per person per year [12]; often, these are unnecessary injections for common symptoms such as fever or fatigue [13]. Although the country has a sufficient supply of syringes [14], high rates of syringe reuse prevail, with 38% of providers likely reusing syringes 2–3 times [15]. Khan et al. (2020) have shown that rural location and longer duration of practice were associated with a higher likelihood of syringe reuse, and both physicians and nonphysicians were equally likely to reuse syringes, despite being aware of the danger of disease transmission [15].

Healthcare workers are at an increased occupational risk of HCV due to needle stick injuries [11]. HCV prevalence is also much higher among persons who inject drugs (PWIDs; AOR: 23.8, CI: 13.0–43.6) [7] and people in prison [16]. In Pakistan, 30.2% of PWIDs have an active infection yet rates of testing are extremely low for this population [17]. Despite this, with a relatively small number (104,804) of active PWIDs in the country, the overall contribution of injecting drug use to HCV incidence is likely much smaller compared to healthcare‐related transmission [18].

Researchers note that, despite a generalised HCV endemic in the country, population risk classification alone explains over 50% of variation in prevalence, meaning that targeted interventions for high‐risk groups would be highly effective [7].

### 1.2 The Health Systems Approach

A health system is a highly complex entity; it consists of all the organisations, resources and people that function primarily to improve health. Using a systems approach to prevent a disease means recognising the multiplicity of elements that interact to impact our outcome of interest and seeking to increase the capacities of the entire health system [19]. Instead of deploying vertical programmes, a systems approach integrates disease‐specific services within currently existing health services. This maximises synergies, removes duplication and promotes cost‐effectiveness [19].

## 2 A Critical Analysis of Pakistan's Health System

Pakistan, located in South Asia, is the world's fifth‐most populous country [20] and is classified as a lower‐middle income country by the World Bank [21]. About 63% of Pakistan's population lives in rural areas, while 37% resides in urban centres [22]. The health system in Pakistan is a mix of public and private providers, with the public sector facing resource constraints and infrastructure deficiencies [23]. Access to healthcare is limited, particularly in rural areas, leading to disparities in health outcomes [24]. Despite these challenges, Pakistan has made strides in improving its health system, including efforts to increase vaccination coverage and address communicable diseases [25].

Researchers note that the devolution of power to provincial governments in Pakistan in 2010 has resulted in health receiving increased priority in terms of government resources, contextualised sector‐wide health planning and measures to regulate healthcare delivery [26]. However, the current infrastructure of provincial hepatitis control programmes is centralised and ineffective, primarily limited to hepatitis clinics at tertiary healthcare facilities [27]. PCR testing for viral hepatitis is available through only a few laboratories, and provincial hepatitis programmes must bear the cost of transporting samples and maintaining sample quality [27]. For patients, the process from test to result can take days or weeks [27], reducing treatment uptake [28]. There are also significant disparities in the availability of HCV treatment services between urban and rural areas. Rural populations often have limited access to specialised care [29].

In 2020, the Prime Minister of Pakistan launched a national program to eliminate HCV by 2030 with a total budget of USD 326 million [30, 31]. However, the subsequent spread of COVID‐19 and changes in leadership led to reallocation of funds, delaying the program's launch [31]. This highlights the need for greater health sector investment and political stability to effectively address the hepatitis C burden in Pakistan [32]. Due to a lack of strategic advocacy, policy makers remain focused on public health challenges associated with lower mortality rates than HCV, such as polio and tuberculosis [27].

### 2.1 Treatment of HCV in Pakistan

Direct‐acting antivirals (DAAs) are a class of medications used to treat HCV. They are more effective than previous medicines, with very high cure rates, often over 95% [33]. Since hepatitis is an infectious disease, treatment of existing cases is important to prevent further spread [19, 34].

**FIGURE 3:**
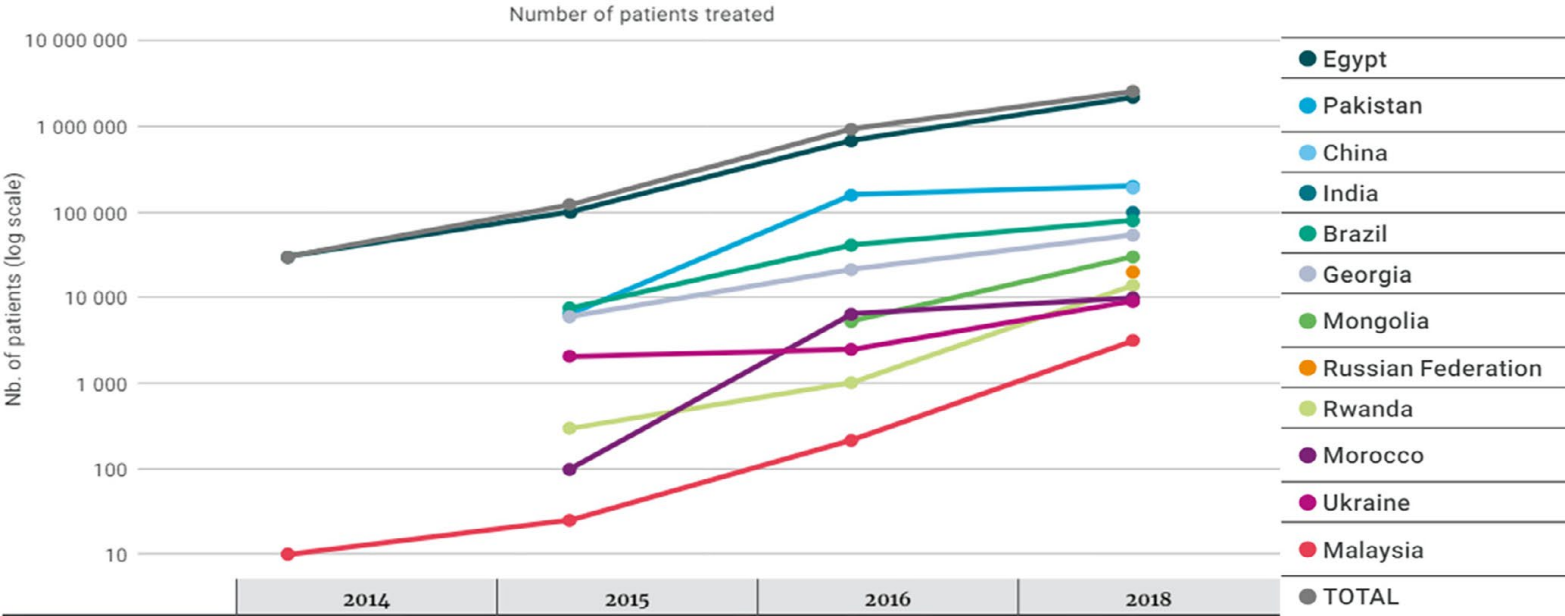
Cumulative number of patients receiving HCV treatment in 12 selected countries—2014 to 2018. In Pakistan, the number has gone from 6,500 in 2015 to 161,000 in 2016, and 200,000 in 2018. Adapted from WHO report [11].

Branded DAAs first became available in Pakistan in 2014. In 2015, the WHO included several DAAs on its essential medicines list, increasing generic competition for these medicines in LMICs [24]. The DAA manufacturer, Gilead, offered generic medicines licences to companies in Pakistan [35], followed by large‐scale local production and a significant drop in prices (Figure 3).

Currently, Pakistan reports one of the lowest treatment costs worldwide, with generic DAAs available for as little as 45 USD per patient [36], compared to the potential cost of 95,000 USD in developed countries [36]. Correspondingly, cure rates have significantly improved. Before 2014, Pakistan reported an average sustained virological response (SVR) of 64%, which increased to 96% in 2017 [37, 38, 39]. However, precise SVR data from government programmes remain scarce due to limited SVR testing [40].

### 2.2 Access to Information on HCV in Pakistan

**FIGURE 4:**
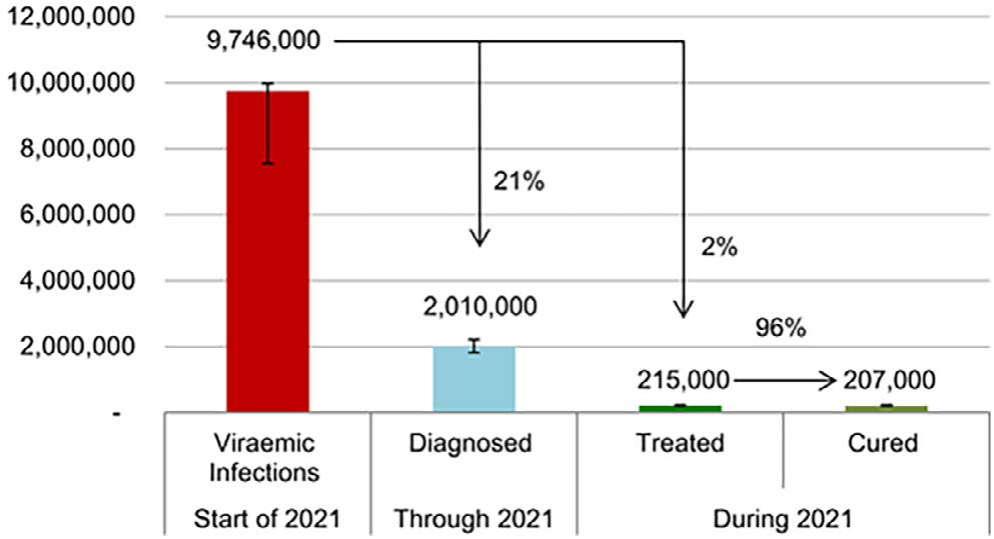
HCV cascade of care for 2021 in Pakistan. Only 21% of viraemic infections were diagnosed in 2021. Adapted from Mooneyhan et al. [40]

Pakistan has several advanced disease surveillance systems [41], such as the Integrated Disease Information Management System (IDIMS), which have been used to successfully prevent the spread of infectious diseases such as COVID‐19 [25]. The pandemic has demonstrated that, despite resource constraints, the country has the surveillance capacity to implement an effective disease prevention strategy [42]. However, researchers have noted that the top‐down disease estimation approach often used may result in an underestimation of the true prevalence of HCV in Pakistan and smaller testing targets set by the government [40]. In Pakistan, the underdiagnosis of viral hepatitis remains a significant barrier to achieving complete elimination [40] (Figure 4).

Other barriers to access of information on HCV in Pakistan include a fragmented health system with multiple stakeholders and levels of care [43], a shortage of skilled personnel to manage and maintain digital health systems [44], and limited access to technologies for digital health systems in rural areas [44]. Currently, all provinces in Pakistan rely on paper‐based data systems, other than the Punjab hepatitis programme, which uses an electronic system to provide real‐time data [31, 45].

### 2.3 Health Workforce and Service Delivery

**FIGURE 5:**
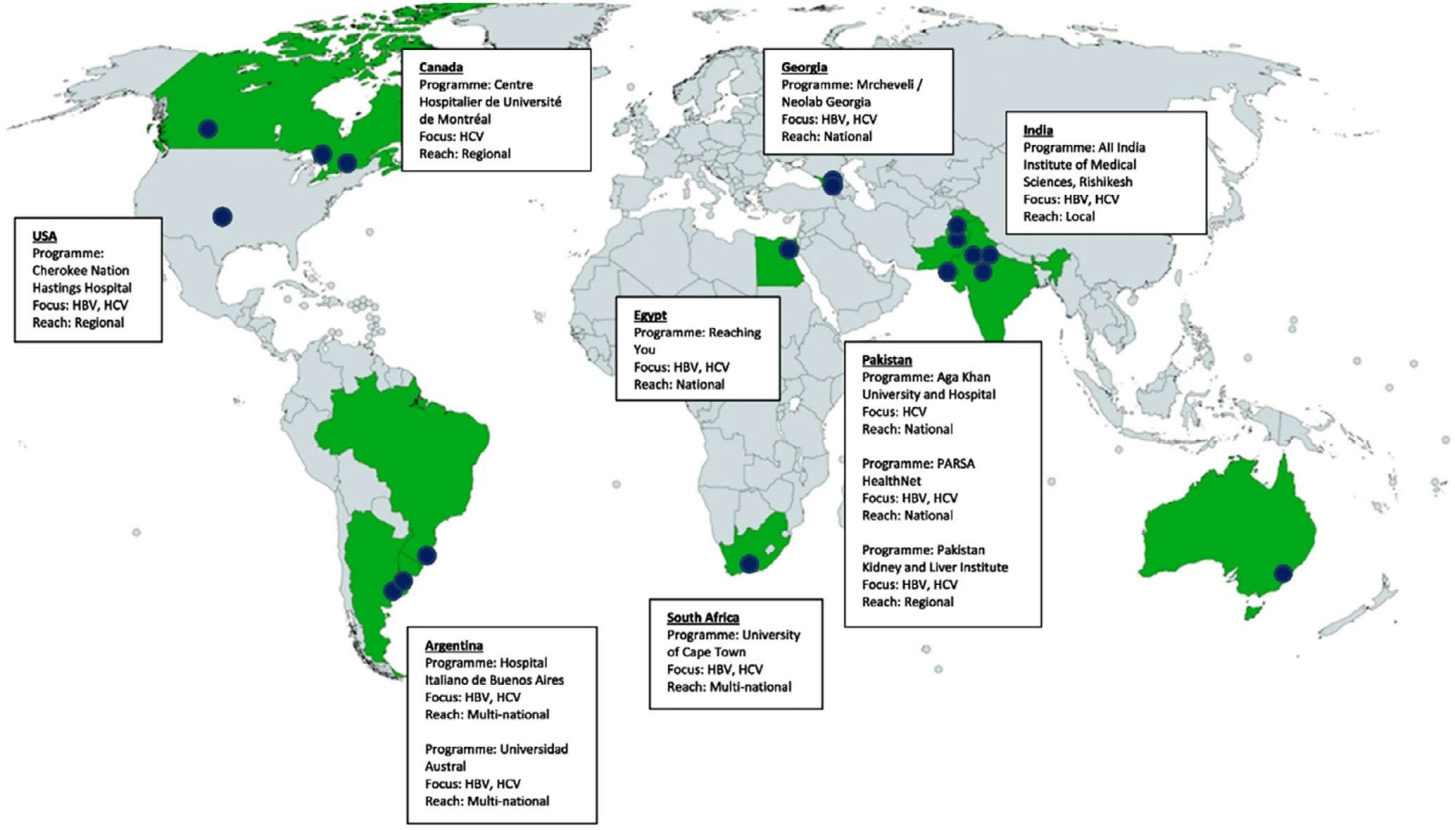
Telementorship programmes affiliated with Project ECHO and focused on HCV. Note the high density of telementorship programmes in Pakistan, many of which have a national reach. Adapted from Corcorran et al. [49]

Health workers in Pakistan acknowledge a simplified treatment algorithm for HCV that requires fewer than two clinic visits [4]. Additionally, the use of a task‐shifting approach in national programmes has addressed the shortage of specialists [46]. General practitioners can initiate treatment for noncirrhotic HCV patients [47]; research shows that task‐shifting to nonspecialists is associated with HCV cure rates comparable to those achieved by specialist care in all studied populations [48]. Furthermore, decentralisation efforts via telementorship in Pakistan have extended HCV care nationwide [49] (Figure 5). These projects have reported high SVR rates across thousands of cases treated [50].

As already noted, the majority of HCV transmission in the country occurs due to unsafe healthcare practices. Healthcare workers in all settings are not adequately aware of the risks of needle stick injuries [51]; informal and private healthcare providers commonly prescribe unnecessary injectable drugs [52], and hospitals and blood banks have poor infection control practices [53, 54].

## 3 HCV Strategy for Pakistan

Pakistan has increased the capacity of PCR testing, electronic health reporting and coordination between its provinces and capital in response to COVID‐19 [42] and is one of the cheapest manufacturers of DAAs [36]. A test‐and‐treat HCV elimination strategy seems plausible for Pakistan, given its success in Egypt‐ another low‐middle‐income country with very high rates of HCV prevalence [55]. The WHO suggests three key intervention targets for eliminating Hepatitis C by 2030: (1) safe surgeries, transfusions and injections; (2) harm reduction; (3) testing and treatment [56].

Pakistan needs to substantially improve access to HCV testing to reach people living with chronic HCV infection, of whom almost 80% remain undiagnosed [40], by operating from existing community and health‐facility‐based services. The following section describes a strategy of integrating HCV diagnostic and treatment services with primary healthcare, thus achieving decentralisation, and reducing healthcare‐associated transmission. With sufficient funding, Pakistan could consider universal HCV screening during primary care clinic visits [57]. This would require significant funding and capacity building at primary care facilities but may be necessary to achieve complete elimination by 2030 [1].

### 3.1 Testing and Treatment

#### 3.1.1 Integration of HCV Services With Primary Healthcare

**FIGURE 6:**
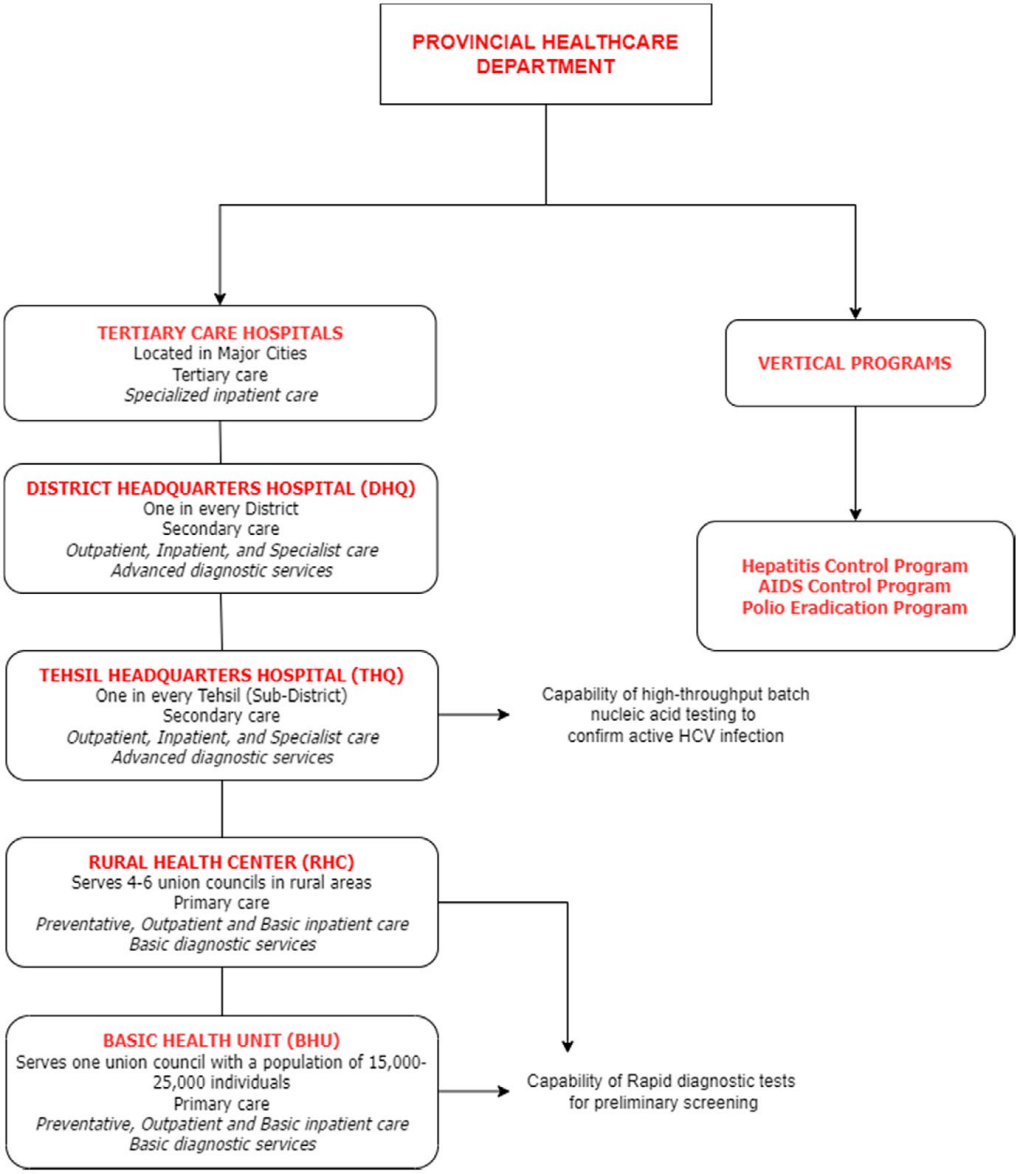
Organization of Pakistan's health system, alongside recommendations for HCV testing. Diagram produced based on content from WHO [58] and Punjab Healthcare Department [59] websites.

At the grassroots level, basic health units (BHUs) and rural health centres (RHCs) serve as primary health facilities in Pakistan and should be equipped to administer rapid diagnostic tests for HCV antibodies to high‐risk patients. Additionally, at least one facility in every subdistrict (Tehsil Headquarters Hospital; THQ) should be capable of performing high‐throughput batch HCV RNA tests for all health facilities in the entire subdistrict to confirm active HCV infection. Figure 6 shows the basic organisation of Pakistan's health delivery system.

Babigumira et al. [60], have determined that, in the Pakistani context, a strategy of batch PCR testing at a centralised laboratory would cost USD 1.67 per person compared to the USD 1.97 per person cost of a point‐of‐care PCR testing approach. However, this analysis may underestimate the full cost burden because it focuses solely on consumables involved in the testing process [60]. Moreover, plasma separation cards for sample collection are easy to use, require minimal training, exhibit high sample stability, and, most importantly, eliminate the need for cold chain storage thus making sample transportation from BHUs to THQs cost‐effective [60]. They reduce the need for patients to travel to distant secondary health facilities. Developed infrastructure could also be utilised for other disease elimination programmes, including those targeting HIV, HBV, *Mycobacterium tuberculosis* , human papillomavirus and SARS‐CoV‐2 [60].

HCV treatment (DAA therapy) should be provided at PHCs; decentralised settings like primary care or community settings have demonstrated comparable hepatitis cure rates to tertiary‐level facilities [48]. The large‐scale decentralisation of hepatitis care should follow a pattern of ‘differentiated’ service delivery through task shifting. All medical officers serving at PHCs should prescribe appropriate DAA therapy to patients with active, uncomplicated HCV infection and medicines should be dispensed from the same health facility [28]. More complicated cases should be referred to the nearest tertiary care hospital. Physicians at PHCs should receive mandatory virtual or in‐person mentorship on HCV management from specialists in the country [49].

#### 3.1.2 Community‐Based Testing

To meet the WHO goal of an 80% reduction in HCV incidence by 2030, Lim et al. [61], propose a strategy of screening 90% of the 2018 population by 2030, with at least 80% of identified cases receiving treatment. The authors recommend targeted screening of high‐risk groups like PWIDs and adults aged more than 30 years old. However, they overlook the fact that over 63% of transmission in Pakistan stems from unsafe healthcare practices [1, 62]. Furthermore, while this approach could reduce incident HCV in Pakistan by 26.5%, it would consume 9% of the country's annual health budget [61].

A more favourable approach may be community‐based screening among high‐risk and high‐prevalence populations. In Pakistan, HCV is more prevalent among older adults [61], people who live in rural areas or peri‐urban areas and individuals who visit informal healthcare providers [63]. Community health workers, such as lady health workers and polio workers, can help raise awareness of HCV [64]. However, integrating HCV testing into their existing initiatives may not be advisable since their primary focus is on young children [65] and women.

Many public–private partnerships have launched microlevel community‐based HCV elimination campaigns in Pakistan. For instance, Médecins Sans Frontières (MSF) implemented two microelimination projects in Karachi with distinct approaches:

1. Baldia: MSF aimed to integrate a decentralised HCV model of care at a PHC run by the Ministry of Health. They encountered significant barriers, including a lack of resources, professionalism and willingness at government‐run facilities, hindering sustainable capacity‐building [64].
2. Machar Colony: MSF independently ran a community‐based vertical HCV program. Health promotion teams went door‐to‐door to raise awareness and invited people to get tested at a local HCV clinic. Despite these efforts, testing rates were low, and only 60% of positive cases initiated treatment [64], whereas Lim et al. recommend at least 80% treatment initiation for effective mass screening programmes [61].

Both experiences highlight the need to build trust within communities before conducting outreach initiatives. Engaging community leaders such as local imams, ensuring transparent communication and prioritising patient‐centred care are critical steps to build this trust.

Another HCV microelimination project took place in the slums of Islamabad through a public–private partnership that screened, tested and treated 50,000 people [31]. Workers conducted doorstep testing and referred positive cases to a public sector PHC dispensary for confirmatory testing and same‐day treatment, resulting in a high treatment initiation rate of 98.5% [31]. For posttreatment SVR testing, community workers followed up with those not tested, leading to a low loss to follow‐up rate of 10% [31]. This project's success could be attributed to the accessibility of communities situated in the capital, the availability of greater resources and an improved community response to public health interventions.

Thus, any community‐based testing initiative should be targeted at high‐risk populations and should aim for same‐day testing and treatment, posttreatment testing through follow‐up, raising awareness and building trust with communities and generating adequate resources and willingness within the organisation to ensure success.

#### 3.1.3 Targeted HCV Screening and Treatment for High‐Risk Groups

In Pakistan, priority populations for HCV screening include people with liver‐related conditions [7], high‐risk clinical populations (populations frequently exposed to blood transfusions or medical injections) [7], and patients undergoing invasive medical procedures [10]. Thus, dialysis centres and surgical wards should postoperatively screen all patients for HCV using low‐cost rapid diagnostic tests. As already mentioned, samples from patients who test positive should be sent using a plasma separation card to the central laboratory at the respective THQ hospital for confirmation of active infection. HCV‐positive patients should receive prompt treatment from their respective site of treatment.

Other priority populations include incarcerated individuals and PWIDs. Oru et al. [48] have shown that full decentralisation and integration of HCV testing and treatment at harm reduction services and prisons is associated with increased linkage to care and treatment and improved treatment uptake. Pakistan should expand its reach of existing harm reduction programmes and implement HCV testing and treatment within these services for PWIDs [48]. Mandatory HCV testing and treatment should also be performed at all prisons in the country [48].

Considering the large population sizes of Sindh and Punjab, Mahmud, Al Kanaani, Abu‐Raddad [7] note that these provinces host nearly 75% of all chronic infections in the country. Therefore, the national government should allocate increased health financing to these provinces' hepatitis control programmes.

#### 3.1.4 Multidisease Diagnostics

The WHO recommends the implementation of multidisease diagnostic platforms for HCV involving the integration of diagnostic capabilities for HCV with those for other prevalent diseases such as human immunodeficiency virus (HIV) and tuberculosis (TB) [24]. For instance, the Abbottm2000 System is a nucleic acid testing platform that can simultaneously test a sample for HIV, HCV, TB, multidrug‐resistant TB, hepatitis B virus, human papillomavirus and at least three other pathogens. The introduction of such shared resources reduces the need to conduct several tests on patients, making diagnosis easier for physicians and more affordable for a greater number of people [24].

### 3.2 Reducing Healthcare‐Associated Transmission

It is unacceptable for the healthcare system to be a source of large‐scale disease transmission. To achieve hepatitis elimination by 2030, the WHO suggests certain service coverage targets, including that all blood donations be screened in a quality‐assured manner, 90% of injections be given with safety‐engineered devices, and at least 300 sterile needles and syringes/PWID/year be distributed.

#### 3.2.1 Safe Injection Practices

Pakistan has a very high rate of intramuscular injections per person per year. Often, these are unnecessary injections for which oral equivalents are available. Research suggests that most healthcare providers are aware of the risks associated with unsafe injections [15]. Hence, it is important to offer guidance on decision‐making for when injectable medicines are warranted and which oral equivalents are available [66]. Health regulatory bodies, hospitals, pharmaceutical companies and pharmacies may all play a role in the dissemination of such information and in ensuring the availability of oral alternatives.

Autodisable (AD) syringes are designed to prevent reuse and are considered an effective strategy for reducing unsafe injection practices. A cost–benefit assessment in India estimated that introducing AD syringes for all medical injections would cost $39–79 per disability‐adjusted life year averted [67], making it a highly cost‐effective intervention [68]. Moreover, Anokhi et al. (2021) found no evidence linking AD syringe design to reuse, adverse events, or blood‐borne virus transmission [69]. Pakistan is one of the 100 countries that exclusively use AD syringes for immunisations [70]. To effectively extend the use of auto‐disable (AD) syringes beyond immunisations, Pakistan should focus on broader healthcare settings, such as therapeutic injections in hospitals and clinics, particularly in rural areas.

#### 3.2.2 Safe Blood Transfusion

Although all blood centres in Pakistan self‐report that they test donated blood for HCV [71], the high incidence of HCV in populations that frequently receive transfusions [7] indicates that there may be shortcomings in this screening system. The findings of the national survey on blood screening systems in Pakistan suggest that frequent interruptions in power supply, the lack of a validation process for consumables, the improper storage of screening kits and the use of rapid test kits in blood banks could be reducing the accuracy of the screening process [71]. Moreover, many blood centres, particularly those in the public sector, do not have a quality assurance policy and most blood banks accord low priority to the implementation of the quality assurance process [71]. Blood transfusion centres should provide services for notification, counselling, referral for care and follow‐up of donors with positive infectious markers for their timely treatment and care, and for minimising the risk of further spread of infection.

Despite past efforts by government and nongovernment organisations, a unified national blood transfusion system based on voluntary, nonrenumerated donations and with high standards of quality assurance is still not in place. With limited resources, the country may consider developing local blood transfusion authorities operating according to national guidelines in all regions of the country. In the capital, the establishment of the Islamabad Blood Transfusion Authority and the subsequent licensing of blood banks has led to significant reform and capacity building [72]. Through an approach of continuous constructive feedback, blood banks in Islamabad have improved their standard of practices, equipment, manpower and financial resource allocations to adhere to the strict minimum licensing criteria [72].

### 3.3 The Challenges Associated With a Health Systems Strengthening Approach

Health system strengthening requires systemwide and sustained effort. The complex nature of the health system makes it difficult to measure success and track specific outcomes. Unlike targeted interventions with immediate results, health system strengthening shows benefits over a longer period. This makes it less appealing to political parties and bilateral donors seeking quick wins. Furthermore, ensuring that reforms are sustainable beyond initial donor funding is challenging. Often, funds can be absorbed into systemic inefficiencies without clear accountability, leading to concerns about wasted resources.

Despite these limitations, it is important to consider the health system strengthening approach as we attempt to eliminate infectious diseases such as HCV. A strengthened health system can facilitate better diagnostic capabilities, more effective treatment delivery, and comprehensive prevention for several blood‐borne pathogens alongside HCV. While the benefits of health system strengthening may not be immediately apparent, it has great potential for creating lasting impact by reducing the burden of disease and improving overall efficiency.

## 4 Conclusion

In conclusion, the high burden of HCV in Pakistan remains a public health challenge of global significance. Key obstacles to HCV elimination include inadequate funding for HCV programmes, a weak service delivery system and unsafe healthcare and community practices leading to transmission. Strategies such as decentralising testing and treatment services, targeting high‐risk populations, and integrating HCV efforts with existing service delivery mechanisms are essential to strengthen the overall health system. With strategic planning, Pakistan can reduce HCV prevalence at comparable rates to other lower‐middle‐income countries like Egypt.

## Conflicts of Interest

The author declares no conflicts of interest.

## Data Availability

Data sharing is not applicable to this article as no new data were created or analyzed in this study.

## References

[1] S. Bajis , T. L. Applegate , J. Grebely , G. V. Matthews , and G. J. Dore , “Novel Hepatitic C Virus (HCV) Diagnosis and Treatment Delivery Systems: Facilitating HCV Elimination by Thinking Outside the Clinic,” Journal of Infectious Diseases 222 (2020): S758–S772. 33245354 10.1093/infdis/jiaa366

[2] A. B. H. Bhatti , F. S. Dar , A. Waheed , K. Shafique , F. Sultan , and N. H. Shah , “Hepatocellular Carcinoma in Pakistan: National Trends and Global Perspective,” Gastroenterology Research and Practice 2016 (2016): 1–10. 10.1155/2016/5942306 PMC4756136 26955390

[3] Geneva: World Health Organization , “Global Health Sector Strategies on HIV,” Viral Hepatitis and the Sexually Transmitted Infections 92 (2021): 005377.

[4] National Hepatitis Elimination Profile , Coaliton for Global Hepatitis Elimination (Pakistan: National Hepatitis Elimination Profile, 2022).

[5] World Health Organization , “15 Million People Affected With Hepatitis B And C in Pakistan: Government Announces Ambitious Plan to Eliminate Hepatitis,” (Geneva: World Health Organization, 2019).

[6] Centre for Disease Control , “Global Viral Hepatitis: Millions of People Are Affected | CDC,” (2024), https://www.cdc.gov/hepatitis/global/index.htm.

[7] S. Mahmud , Z. Al Kanaani , and L. J. Abu‐Raddad , “Characterization of the Hepatitis C Virus Epidemic in Pakistan,” BMC Infectious Diseases 19, no. 1 (2019): 1–11. 31521121 10.1186/s12879-019-4403-7 PMC6744714

[8] Pakistan Medical Research Council , “Hepatitis B and C Survey Pakistan 2007–2008 | Pakistan Health Knowledge Hub,” Pakistan Journal of Medical Sciences 3 (2008): 1003–1017, https://phkh.nhsrc.pk/knowledge‐article/hep‐b‐and‐hep‐c‐survey‐pakistan‐phrc‐2007‐08.

[9] K. Alaei , M. Sarwar , and A. Alaei , “The Urgency to Mitigate the Spread of Hepatitis C in Pakistan Through Blood Transfusion Reform,” International Journal of Health Policy and Management 7, no. 3 (2018): 207–209. 29524949 10.15171/ijhpm.2017.120 PMC5890065

[10] S. Savul , F. K. Lalani , A. Ikram , M. A. Khan , M. A. Khan , and J. Ansari , “Infection Prevention and Control Situation in Public Hospitals of Islamabad,” Journal of Infection in Developing Countries 14, no. 9 (2020): 1040–1046. 33031094 10.3855/jidc.12779

[11] A. Bosan , H. Qureshi , K. M. Bile , I. Ahmad , and R. Hafiz , “A Review of Hepatitis Viral Infections in Pakistan,” Journal of the Pakistan Medical Association 60, no. 12 (2010): 1045–1058. 21381562

[12] N. Z. Janjua , S. Akhtar , and Y. J. F. Hutin , “Injection Use in Two Districts of Pakistan: Implications for Disease Prevention,” International Journal for Quality in Health Care 17, no. 5 (2005): 401–408. 15883127 10.1093/intqhc/mzi048

[13] S. A. Ali , R. M. J. Donahue , H. Qureshi , and S. H. Vermund , “Hepatitis B and Hepatitis C in Pakistan: Prevalence and Risk Factors,” International Journal of Infectious Diseases 13, no. 1 (2009): 9–19. 18835208 10.1016/j.ijid.2008.06.019 PMC2651958

[14] A. A. Khan , M. Saleem , H. Qureshi , R. Jooma , and A. Khan , “Comparison of Need and Supply of Syringes for Therapeutic Injections in Pakistan,” Journal of the Pakistan Medical Association 62, no. 11 (2012): 1149–1153. 23866401

[15] A. A. Khan , A. Altaf , H. Qureshi , M. Orakzai , and A. Khan , “Reuse of Syringes for Therapeutic Injections in Pakistan: Rethinking Determinants,” Eastern Mediterranean Health Journal 26, no. 3 (2020): 283–289. 32281637 10.26719/emhj.19.028

[16] A. M. Kazi , S. A. Shah , C. A. Jenkins , B. E. Shepherd , and S. H. Vermund , “Risk Factors and Prevalence of Tuberculosis, Human Immunodeficiency Virus, Syphilis, Hepatitis B Virus, and Hepatitis C Virus Among Prisoners in Pakistan,” International Journal of Infectious Diseases 14S3, no. SUPPL. 3 (2010): e60–e66. 10.1016/j.ijid.2009.11.012 PMC2905608 20189863

[17] B. Hajarizadeh , A. Kairouz , S. Ottaviano , et al., “Global, Regional, and Country‐Level Coverage of Testing and Treatment for HIV and Hepatitis C Infection Among People Who Inject Drugs: A Systematic Review,” Lancet Global Health 11, no. 12 (2023): e1885–e1898. 37973339 10.1016/S2214-109X(23)00461-8

[18] Z. Al Kanaani , S. Mahmud , S. P. Kouyoumjian , and L. J. Abu‐Raddad , “The Epidemiology of Hepatitis C Virus in Pakistan: Systematic Review and Meta‐Analyses,” Royal Society Open Science 5, no. 4 (2018): 180257. 29765698 10.1098/rsos.180257 PMC5936963

[19] Geneva: World Health Organization , “Global Health Sector Stategies on, Respectively, HIV, Viral Hepatitis and Sexual Transmitted Infections for the Period 2022–2030,” Brazilian Dental Journal 33, no. 1 (2022): 1–12.

[20] Government of Pakistan , “Population Census | Pakistan Bureau of Statistics,” (2018), https://www.pbs.gov.pk/content/population‐census.

[21] World Bank , “World Bank Country and Lending Groups—World Bank Data,” (2024), https://datahelpdesk.worldbank.org/knowledgebase/articles/906519‐world‐bank‐country‐and‐lending‐groups.

[22] United Nations , “World Urbanization Prospects—Population Division—United Nations,” (2018), https://population.un.org/wup/.

[23] U. Afzal and A. Yusuf , “The State of Health in Pakistan: An Overview,” Lahore Journal of Economics special edition vol. 18 (2013): 233–247, https://papers.ssrn.com/abstract=2476075.

[24] Organization WHO , “Accelerating Access to Hepatitis C Diagnostics and Treatment Overcoming Barriers in Low‐and Middle‐Income Countries,” Global Process Report 2021, Vol. 1 (Geneva: World Health Organization, 2020), apps.who.int.

[25] F. Emmanuel , A. Hassan , A. Ahmad , and T. E. Reza , “Pakistan's COVID‐19 Prevention and Control Response Using the World Health Organization's Guidelines for Epidemic Response Interventions,” Cureus 15, no. 1 (2023): 34480. 10.7759/cureus.34480 PMC9982052 36874693

[26] S. A. Zaidi , M. Bigdeli , E. V. Langlois , et al., “Health Systems Changes After Decentralisation: Progress, Challenges and Dynamics in Pakistan,” BMJ Global Health 4, no. 1 (2019): e001013. 10.1136/bmjgh-2018-001013 PMC6357909 30805206

[27] N. Ali and A. Charting , New Course to Hepatitis Elimination in Pakistan, (Geneva: Health Policy Watch, 2024).

[28] Z. Mohamed , D. Al‐Kurdi , M. Nelson , et al., “Time Matters: Point of Care Screening and Streamlined Linkage to Care Dramatically Improves Hepatitis C Treatment Uptake in Prisoners in England,” International Journal on Drug Policy 1, no. 75 (2020): 102608. 10.1016/j.drugpo.2019.102608 31759307

[29] S. Siddiqi , M. S. Khan , N. Rizvi , et al., “Are Rural Hospitals in Pakistan Responding to the Global Surgery Movement? An Analysis of the Gaps, Challenges and Opportunities,” World Journal of Surgery 44, no. 4 (2020): 1045–1052. 31848676 10.1007/s00268-019-05327-x

[30] H. Qureshi , Prime Minister's Programme for the Elimination of Hepatitis C, (Islamabad: Ministry of Health, 2020).

[31] H. Qureshi , H. Mahmood , A. Sabry , and J. Hermez , “Barriers and Strategies for Hepatitis B and C Elimination in Pakistan,” Journal of Infectious Diseases 228, no. Supplement_3 (2023): S204–S210. 37703344 10.1093/infdis/jiad022

[32] D. K. Ciccone , T. Vian , L. Maurer , and E. H. Bradley , “Linking Governance Mechanisms to Health Outcomes: A Review of the Literature in Low‐ and Middle‐Income Countries,” Social Science & Medicine 1, no. 117 (2014): 86–95. 10.1016/j.socscimed.2014.07.010 25054281

[33] M. Martinello , S. Naggie , J. K. Rockstroh , and G. V. Matthews , “Direct‐Acting Antiviral Therapy for Treatment of Acute and Recent Hepatitis C Virus Infection: A Narrative Review,” Clinical Infectious Diseases 77, no. Supplement_3 (2023): S238–S244. 37579203 10.1093/cid/ciad344

[34] H. H. Ayoub and L. J. Abu‐Raddad , “Treatment as Prevention for Hepatitis C Virus in Pakistan: Mathematical Modelling Projections,” BMJ Open 9, no. 5 (2019): e026600. 10.1136/bmjopen-2018-026600 PMC6537971 31133586

[35] “Access Partnerships | Gilead,” (2024), https://www.gilead.com/purpose/medication‐access/global‐access/access‐partnerships.

[36] V. S. Balakrishnan , “Essential Medicines for Hepatitis C: At What Price?,” Lancet Infectious Diseases 17, no. 9 (2017): 904–905. 28845797 10.1016/S1473-3099(17)30465-6

[37] Z. Ullah , S. Z. Khan , H. Lodhi , H. Khan , R. Hidayat , and M. Ahmed , “Efficacy of Sofosbuvir and Daclatasvir in Achieving the End Treatment Response and Sustained Viral Response in Patients Infected With Hepatitis C Virus Genotype 3,” Pakistan Armed Forces Medical Journal 72, no. 3 (2022): 1074–1077.

[38] S. Kazmi , H. Farooqi , U. Sohail , S. H. Zaidi , N. Majeed , and S. Firdus , “Sustained Virological Response (SVR) and Safety of Two Direct Acting Anti‐Viral (DAA) Combination Therapies in Chronic Hepatitis‐C Infected Patients of Lahore, Pakistan. A Randomized Controlled Trial: SVR and DAA Therapies in Hepatitis‐C Infected Patients,” Pakistan Journal of Medical Sciences 3, no. 6 (2022): 135–139.

[39] S. Mushtaq , A. Mansoor , M. Umar , A. Khan , S. Siddiqi , and S. Manzoor , “Direct‐Acting Antiviral Agents in the Treatment of Chronic Hepatitis C—Real‐Life Experience From Clinical Practices in Pakistan,” Journal of Medical Virology 92, no. 12 (2020): 3475–3487. 32129507 10.1002/jmv.25745

[40] E. Mooneyhan , H. Qureshi , H. Mahmood , et al., “Hepatitis C Prevalence and Elimination Planning in Pakistan, a Bottom‐Up Approach Accounting for Provincial Variation,” Journal of Viral Hepatitis 30, no. 4 (2023): 345–354. 36650932 10.1111/jvh.13802

[41] W. Bilal , K. Qamar , S. Abbas , A. Siddiqui , and M. Y. Essar , “Infectious Diseases Surveillance in Pakistan: Challenges, Efforts, and Recommendations,” Annals of Medicine and Surgery 1 (2022): 78. 10.1016/j.amsu.2022.103838 PMC9207098 35734665

[42] N. Noreen , S. A. U. Rehman , I. Naveed , S. U. K. Niazi , and I. B. Furqan , “Pakistan's COVID‐19 Outbreak Preparedness and Response: A Situational,” Analysis 19, no. 6 (2021): 605–615, https://home.liebertpub.com/hs. 10.1089/hs.2021.0006 34762516

[43] T. Tamrat , S. Chandir , K. Alland , et al., “Digitalization of Routine Health Information Systems: Bangladesh, Indonesia, Pakistan,” Bulletin of the World Health Organization 100, no. 10 (2022): 590–600. 36188022 10.2471/BLT.22.287816 PMC9511663

[44] M. Malik , A. F. Kazi , and A. Hussain , “Adoption of Health Technologies for Effective Health Information System: Need of the Hour for Pakistan,” PLoS One 16, no. 10 (2021): e0258081. 34618842 10.1371/journal.pone.0258081 PMC8496784

[45] S. S. Keyani , A. A. Mumtaz , and A. Ahmad , “Hepatitis Surveillance System for Rural Pakistan Through Web and Mobile Based Technologies. 2014 11th Annu High Capacit Opt Networks Emerging/Enabling Technol (Photonics Energy),” HONET‐PfE 2014, no. 2 (2014): 190–194.

[46] A. Yusufzai , Patients Suffer Owing to Lack of Gastro Wards in KP DHQ Hospitals—Pakistan (Dawn, 2023).

[47] C. E. Boeke , C. Adesigbin , C. Agwuocha , et al., “Initial Success From a Public Health Approach to Hepatitis C Testing, Treatment and Cure in Seven Countries: The Road to Elimination,” BMJ Global Health 5, no. 12 (2020): e003304. 10.1136/bmjgh-2020-003767 PMC7745326 33328200

[48] E. Oru , A. Trickey , R. Shirali , S. Kanters , and P. Easterbrook , “Decentralisation, Integration, and Task‐Shifting in Hepatitis C Virus Infection Testing and Treatment: A Global Systematic Review and Meta‐Analysis,” Lancet Global Health 9, no. 4 (2021): e431–e445. 33639097 10.1016/S2214-109X(20)30505-2 PMC7966682

[49] M. A. Corcorran , K. Thornton , B. Struminger , P. Easterbrook , and J. D. Scott , “Training the Healthcare Workforce: The Global Experience With Telementorship for Hepatitis B and Hepatitis C,” BMC Health Services Research 23, no. 1 (2023): 1–15. 37533025 10.1186/s12913-023-09849-y PMC10394928

[50] Parsa Trust , “PARSA HealthNet ECHO,” (2024), https://parsatrust.pk/parsa‐healthnet‐echo/.

[51] A. R. Qazi , F. A. Siddiqui , S. Faridi , et al., “Comparison of Awareness About Precautions for Needle Stick Injuries: A Survey Among Health Care Workers at a Tertiary Care Center in Pakistan,” Patient Safety in Surgery 10, no. 1 (2016): 1–6. 27610201 10.1186/s13037-016-0108-7 PMC5015332

[52] S. Siddiqi , S. Hamid , G. Rafique , et al., “Prescription Practices of Public and Private Health Care Providers in Attock District of Pakistan,” International Journal of Health Planning and Management 17, no. 1 (2002): 23–40. 11963441 10.1002/hpm.650

[53] S. Bibi , T. Siddiqui , S. Jafry , and W. Ahmed , “Infection Control Practices in Blood Banks of Pakistan,” Eastern Mediterranean Health Journal 25, no. 5 (2019): 331–340. 31364758 10.26719/emhj.18.051

[54] U. R. Khan , N. Z. Janjua , S. Akhtar , and J. Hatcher , “Case–Control Study of Risk Factors Associated With Hepatitis C Virus Infection Among Pregnant Women in Hospitals of Karachi‐Pakistan,” Tropical Medicine & International Health 13, no. 6 (2008): 754–761. 18384475 10.1111/j.1365-3156.2008.02075.x

[55] G. Esmat , T. Elbaz , A. Elsharkawy , M. Abdullah , and M. El Kassas , “Emerging From the Screening of 57 Million Citizens and Treating 4 Million Patients: Future Strategies to Eliminate Hepatitis C From Egypt,” Expert Review of Anti‐Infective Therapy 18, no. 7 (2020): 637–642. 32302245 10.1080/14787210.2020.1758065

[56] World Health Organization , “Combating hepatitis B and C to reach elimination by 2030,” (2016).

[57] L. M. Hagan and R. F. Schinazi , “Best Strategies for Global HCV Eradication,” Liver International 33, no. 1 (2013): 68–79. 23286849 10.1111/liv.12063 PMC4110680

[58] World Health Organization , “WHO EMRO | Health Service Delivery | Programmes | Pakistan,” (2024), https://www.emro.who.int/pak/programmes/service‐delivery.html.

[59] Government of the Punjab , “Primary & Secondary Health Care Department,” 2024), https://pshealthpunjab.gov.pk/Home/Services.

[60] J. B. Babigumira , J. K. Karichu , S. Clark , et al., “Assessing the Potential Cost‐Effectiveness of Centralised Versus Point‐of‐Care Testing for Hepatitis C Virus in Pakistan: A Model‐Based Comparison,” BMJ Open 13, no. 5 (2023): e066770. 10.1136/bmjopen-2022-066770 PMC10163545 37142306

[61] A. G. Lim , J. G. Walker , N. Mafirakureva , et al., “Effects and Cost of Different Strategies to Eliminate Hepatitis C Virus Transmission in Pakistan: A Modelling Analysis,” Lancet Global Health 8, no. 3 (2020): e440–e450. 32087176 10.1016/S2214-109X(20)30003-6 PMC7295205

[62] Global Burden of Disease Collaborative Network , Global Burden of Disease Study 2019. Results 2019 (Seattle, WA: Institute for Health Metrics and Evaluation, 2020).

[63] M. Mansoor , W. A. de Glanville , R. Alam , et al., “Prevalence and Risk Factors for Hepatitis C Virus Infection in an Informal Settlement in Karachi, Pakistan,” PLOS Globel Public Health 3, no. 9 (2023): e0002076. 10.1371/journal.pgph.0002076 PMC10511086 37729129

[64] E. Rasheed , Evaluation of the Hepatitis C Strategy in MSF's Project in Karachi, Pakistan | MSF Intersectional Evaluation Group (Stockholm, Sweden: Médecins Sans Frontières, 2023), https://evaluation.msf.org/evaluation‐report/evaluation‐hepatitis‐c‐strategy‐msfs‐project‐karachi‐pakistan.

[65] W. Jafri , N. Jafri , J. Yakoob , et al., “Hepatitis B and C: Prevalence and Risk Factors Associated With Seropositivity Among Children in Karachi, Pakistan,” BMC Infectious Diseases 6, no. 1 (2006): 1–10. 16792819 10.1186/1471-2334-6-101 PMC1539007

[66] C. Gore , J. V. Lazarus , R. J. J. Peck , I. Sperle , and K. Safreed‐Harmon , “Unnecessary Injecting of Medicines Is Still a Major Public Health Challenge Globally,” Tropical Medicine & International Health 18, no. 9 (2013): 1157–1159. 23876226 10.1111/tmi.12151

[67] S. Reid , “Estimating the Burden of Disease From Unsafe Injections in India: A Cost‐Benefit Assessment of the Auto‐Disable Syringe in a Country With Low Blood‐Borne Virus Prevalence,” Indian Journal of Community Medicine 37, no. 2 (2012): 89–94. 22654281 10.4103/0970-0218.96093 PMC3361807

[68] R. Laxminarayan , J. Chow , and S. A. Shahid‐Salles , Intervention Cost‐Effectiveness: Overview of Main Messages, (Washington, DC: The International Bank for Reconstruction and Development, 2006). 21250358

[69] A. Ali Khan , M. Munir , F. Miraj , et al., “Examining Unsafe Injection Practices Associated With Auto‐Disable (AD) Syringes: A Systematic Review,” Human Vaccines & Immunotherapeutics 17, no. 9 (2021): 3247–3258. 33989509 10.1080/21645515.2021.1911514 PMC8381785

[70] J. Fritz , E. Griffin , R. Hammack , T. Herrick , and C. Jarrahian , “Syringes Must Be Prioritized Globally to Ensure Equitable Access to COVID‐19 and Other Essential Vaccines and to Sustain Safe Injection Practices,” Human Vaccines & Immunotherapeutics 18, no. 7 (2022): 2077580. 35648471 10.1080/21645515.2022.2077580 PMC9891668

[71] H. Zaheer and U. Waheed , “National Baseline Survey on Monitoring and Evaluation of Blood Screening Systems in Pakistan,” Journal of Blood Disorders & Transfusion 6, no. 2 (2015): 1000265.

[72] H. Zaheer and U. Waheed , “Impact of Regulation of Blood Transfusion Services in Islamabad, Pakistan,” Global Journal of Transfusion Medicine 1, no. 1 (2016): 29.

